# The *International Health Partnership Plus*: rhetoric or real change? Results of a self-reported survey in the context of the 4^th ^high level forum on aid effectiveness in Busan

**DOI:** 10.1186/1744-8603-8-13

**Published:** 2012-05-31

**Authors:** Tim Shorten, Martin Taylor, Neil Spicer, Sandra Mounier-Jack, David McCoy

**Affiliations:** 1 Lymehouse Studios, 38 Georgiana Street, London, NW1 0EB, UK; 2 Universal House, 1-2 Queen’s Parade Place, Bath, BA1 2NN, UK; 3 The London School of Hygiene and Tropical Medicine, 15-17 Tavistock Place, London, WC1H 9SH, UK; 4 McCoy D, University College London, Gower Street, London, WC1E 6BT, UK

**Keywords:** Aid effectiveness, Accountability, Global health policy, Monitoring and evaluation, International health partnership

## Abstract

**Background:**

The Paris Declaration on Aid Effectiveness, which provides an international agreement on how to deliver aid, has recently been reviewed by the Organization for Economic Co-operation and Development (OECD). Health sector aid effectiveness is important, given the volume of financial aid and the number of mechanisms through which health assistance is provided. Recognizing this, the international community created the International Health Partnership (IHP+), to apply the Paris Declaration to the health sector. This paper, which presents findings from an independent monitoring process (*IHP+Results*), makes a valuable contribution to the literature in the context of the recent 4^th^ High Level Forum on Aid Effectiveness in Busan, Korea.

**Methods:**

*IHP+Results* monitored commitments made under the IHP + using an agreed framework with twelve measures for IHP + Development Partners and ten for IHP + recipient country governments. Data were collected through self-administered survey tools. *IHP+Results* analyzed these data, using transparent criteria, to produce Scorecards as a means to highlight progress against commitments and thereby strengthen mutual accountability amongst IHP + signatories.

**Results:**

There have been incremental improvements in the strengthening of national planning processes and principles around mutual accountability. There has also been progress in Development Partners aligning their support with national budgets. But there is a lack of progress in the use of countries’ financial management and procurement systems, and in the integration of duplicative performance reporting frameworks and information systems.

**Discussion and Conclusions:**

External, independent monitoring is potentially useful for strengthening accountability in health sector aid. While progress in strengthening country ownership, harmonisation and alignment seems evident, there are ongoing challenges. In spite of some useful findings, there are limitations with IHP + monitoring that need to be addressed. This is not surprising given the challenge of rigorously monitoring Development Partners across multiple recipient countries within complex global systems. The findings presented here suggest that the health sector is ahead of the game – in terms of having an established mechanism to promote alignment and harmonisation, and a relatively advanced monitoring framework and methods. But to capitalise on this, IHP + signatories should: a) reaffirm their commitments to the IHP+; b) actively embrace and participate in monitoring and evaluation processes; and c) strengthen in-country capacity notably amongst civil society organizations.

## Background

On the 5th of September 2011, the International Health Partnership *and related initiatives* (IHP+) celebrated its fourth anniversary. In 2007 26 signatories signed the IHP + Global Compact with a commitment to ‘work effectively together with renewed urgency to build sustainable health systems and improved health outcomes’. The Compact committed signatories to making concrete the 2005 Paris Declaration of Aid Effectiveness in the field of health: to improve aid coordination; increase the predictability of donor aid flows; work towards strengthened country health systems; and renew commitment to mutual accountability and transparency. Importantly, it also committed to independent monitoring of IHP + signatories’ efforts to improve aid effectiveness (see below for more details). Today the IHP + enjoys an expanded constituency (Table 1), and has demonstrated its commitment to independent assessment and accountability by commissioning in 2009 a partnership known as *IHP+Results* to independently monitor progress against agreed indicators.

**Table 1:** IHP + Signatories

	**Original signatories**	**New signatories**
Bilateral agencies	Canada; France; Germany; Italy; Netherlands; Norway; Portugal;UK.	Australia, Belgium; Spain; Finland; Sweden
Multilateral agencies	African Development Bank (AfDB); European Commission (EC); UNAIDS; UNDP; UNFPA; UNICEF; WHO; World Bank.	OECD-DAC; ILO
GHPs	GAVI Alliance (GAVI); Global Fund to Fight AIDS, TB and Malaria (Global Fund)	
Private Foundations	The Bill & Melinda Gates Foundation	
Country governments	Burundi; Cambodia; Ethiopia; Kenya; Mozambique; Nepal; Zambia	Burkina Faso; Ghana; Mali; Madagascar; Niger; Nigeria; Cameroon; Chad; Democratic Republic of Congo (DRC); Djibouti; El Salvador; Mauritania; Pakistan; Rwanda; Senegal; Sierra Leone; Sudan; Togo; Uganda; Vietnam

On the 29^th^ November 2011 senior representatives of the global aid industry met at the Fourth High Level Forum in Busan, South Korea, to review their collective efforts in improving the effectiveness of aid.^a^ This paper, which presents findings from *IHP+Results* monitoring in 2010, makes a timely and valuable contribution to the literature in the context of Busan.

### **Key features of the International Health Partnership (IHP+)**

IHP + Global Compact defines commitments following the Paris principles of:

• National ownership

• Alignment with national systems

• Harmonization between agencies

• Managing for results

• Mutual accountability

The intended benefits for developing countries are [1]: 

• Improved results through better use of existing funds

• Improved harmonization and alignment of aid to reduce fragmentation and transaction costs

• Improved coordination between country governments and Development Partners

• Strengthened mutual accountability and transparency, progressively involving all stakeholders in existing national planning and monitoring processes

• Long-term predictable financing for strengthening health systems

• Stronger government leadership in sector coordination

How does IHP + work?

IHP + encourages increased support for one national health plan through

• Support to **national sector planning processes**

• Creating greater confidence in national plans by encouraging **joint assessment** of their strengths and weaknesses

• More unified modalities for partner support to the plan, with the development or strengthening of **country compacts**[2]

• **One results monitoring framework** to track plan implementation

• Greater mutual accountability by **monitoring progress against compact commitments**[3]

The day-to-day work of the Partnership is facilitated through a Core Team jointly hosted by the World Health Organization (WHO) and the World Bank [4], reporting to the IHP + Executive Team and the IHP + Scaling Up Reference Group (SuRG).

The multiple problems of aid – generally and for health specifically - are long recognised and have been widely reported. Although the last two decades have seen a substantial increase in development assistance for health from $5B in 1990 to $21.8B in 2007 [5], all too commonly aid for health programs is not used effectively, and in some cases has negative consequences for recipient countries [6-19]. Lack of predictability is an important problem, both in terms of timely disbursement of aid, and the tendency for donors to make short-term financial commitments. It is damaging not only because it reduces the value of aid by 15-20% [6], but it can also increase fiscal and monetary instability in recipient countries, which can heighten inflation [7]. Increases in health aid occurred in parallel with a proliferation of global health actors, which has heightened concerns about poor harmonization, as well as limited alignment between donor programs and country priorities. Dodd et al. [8] state: ‘… *there are now well over a 100 major international organizations involved in health, far more than in any other sector, and literally hundreds of channels for delivering health aid*’. Most donors have their own approaches and procedures that place substantial demands on fragile recipient country health systems [9-12]. Lack of accountability and transparency are also critical shortcomings: many development agencies reveal little about how and why decisions are made, and are more accountable to donors and tax payers in high income countries than to recipients or beneficiaries in low income countries [13]. Many commentators further suggest that donor government national security, economic and foreign policy interests drive and explain donor behaviour rather than the health needs of people in low-income countries [14,15]. It is therefore not surprising to see a lack of ownership of health programs from recipient country governments, which undermines government accountability to their own populations.

A multitude of national and international declarations and initiatives have been launched with the objective of improving the effectiveness of development assistance for health programs [15,16] (see Table 2). The principles articulated in the Paris Declaration on Aid Effectiveness, which was endorsed by over 100 signatories, have galvanized commitments from both Development Partners and recipient governments to work towards improving aid effectiveness. The Paris agenda was enthusiastically adopted by the health sector with the launch of the IHPIHP +.

**Table 2:** Major aid effectiveness declarations, initiatives and processes

	
1980s	National AIDS Commissions (NACs) or equivalent
1997	Sector Wide Approaches (SWAPs)
	Poverty Reduction Strategies
2001	Global Fund Country Coordination Mechanisms
2002	Monterrey Consensus on Financing for Development
2003	Rome Declaration of Harmonisation
2004	The ‘Three Ones’ Principles
2004	Joint Marrakech Memorandum on Managing for Results
2005	Paris Declaration on Aid Effectiveness
2005	Global Task Team on Improving AIDS Coordination among Multilateral Institutions and International Donors
2006	UN’s ‘Delivering as One’
2007	Health 8 Agencies (H8)
2007	Global Implementation Support Team
2007	Global Campaign for the Health MDGs
2007	International Health Partnership (IHP+) Global Compact
2008/9	International Health Partnership (IHP+) Country Compacts
2008	Health Systems Funding Platform
2008	Accra Third High Level Forum on Aid Effectiveness
2011	Global Fund National Strategy Applications (NSAs)
2011	Busan Fourth High Level Forum on Aid Effectiveness

From the start, there have been concerns expressed that the IHP + will turn out to be more rhetoric than real: the latest in a long line of failed declarations and initiatives to reduce some of the damaging effects of uncoordinated and misdirected aid. This paper sheds light on this question by critically assessing the progress made by the IHP+. It draws on the monitoring conducted by *IHP+Results* in 2010 [17] (the methods are described below), and discusses it in relation to broader aid effectiveness monitoring. Below we examine the extent to which Development Partners and recipient governments’ IHP + commitments are being demonstrated through changes in the ways they work; we reflect on a methodology used to monitor the implementation of IHP + commitments, and raise questions and recommendations that remain relevant both in the aftermath of the 4^th^ High Level Forum on Aid Effectiveness and to the future of the IHP +.

## Methods

*IHP+Results* is an independent north–south consortium of research and advocacy organisations, mandated by IHP + signatories to provide an annual assessment of the implementation of the commitments set out in the IHP + Global Compact [18]. The reporting framework used by *IHP+Results* was agreed by all IHP + signatories. A set of ten measures for IHP + country governments and twelve for Development Partners were agreed, based closely on the Paris Declaration indicators and selected to track progress against the results expected. Targets for each indicator were agreed, drawing on Paris Declaration targets where applicable; and finalised by *IHP+Results* in conjunction with the IHP + Working Group on Mutual Accountability (consisting of a time-limited group of IHP + signatories mandated to agree to a monitoring framework for use by *IHP+Results*). A timeframe for data collection and analysis, including presentation of results through ‘Scorecards’ (described below), was also agreed by IHP + signatories. Participation in the monitoring process - consisting of agreement to provide data against the agreed framework, and for a Scorecard to be produced - was on a voluntary basis. Twenty-five IHP + signatories opted to participate in 2010 (see below).

**Participants in *IHP+Results* 2010 monitoring**

10 IHP ± country governments: Burkina Faso, Burundi, Djibouti, DRC, Ethiopia, Mali, Mozambique, Nepal, Niger and Nigeria

15 Development Partners: AusAID, Belgium, EC, GAVI, Global Fund, Netherlands, Norway, Spain, Sweden, UK, UNAIDS, UNFPA, UNICEF, WHO and World Bank

To collect data, *IHP+Results* used a self-administered survey that would be completed by both Development Partners and recipient governments. The survey drew far as possible on the OECD/DAC guidance for the 2011 Survey on Monitoring the Paris Declaration. Survey tools and guidance documentation were made available in English and French.

Participants had six weeks to complete the survey and. Development Partners reported data for those countries in which they considered *themselves* to be active, from amongst the ten countries participating in the survey. The *IHP+Results* team provided support to survey participants throughout the data collection process.

IHP+Results reviewed the completed survey tools and cleaned the data to maintain consistent application of guidance on key terms and definitions. Data reported by Development Partners for each recipient country were aggregated using a weighted method (see below) for the purposes of providing an agency-level Scorecard.

Formula for aggregating data presented in Partner Scorecard ratings

Aggregate Numerator ÷ Aggregate Denominator = Result

*Example:*

(1) $$\frac{\text{Numerator }\left(\text{country}1\right)\text{ + Numerator }\left(\text{country }2\right)}{\text{Denominator }\left(\text{country}1\right)\text{ + Denominator }\left(\text{country }2\right)}\text{ = Result}$$

Agreed, transparent criteria (see Table 3) were applied to aggregated data to produce a draft Scorecard for each signatory. Where insufficient data were available to enable a rating, a ‘question mark’ rating was produced. Two data points were collected: 2009 (as the latest available year) and baseline data (for the period 2005–2007, depending on the most recent data that each participating signatory could provide).

**Table 3:** Criteria for scorecard ratings

		
Target achieved	Target not reached, but there is demonstrated evidence of progress towards reaching the target.	No demonstrated evidence of progress towards the target.

Scorecards for Development Partners were based on aggregated data, which could mask variable performance across different countries. In this paper we report on overall aggregated data. However, disaggregated (country-level) ratings were also provided (http://www.ihpresults.net) so that the performance of each Development Partner could be viewed on a country-by-country basis.

Scorecards were shared with all participating signatories to ensure that the interpretation and presentation of data were accurate. The data and Scorecards were analysed by *IHP+Results* and an independent synthesis of the findings, with conclusions and recommendations based on this evidence, was presented in *IHP+Results* 2010 Annual Performance Report. A full description of *IHP+Results*’ methodology, including a discussion of observed limitations is available at http://www.ihpresults.net/how/methodology

## Results

### Overview of findings

**Figure 1:**
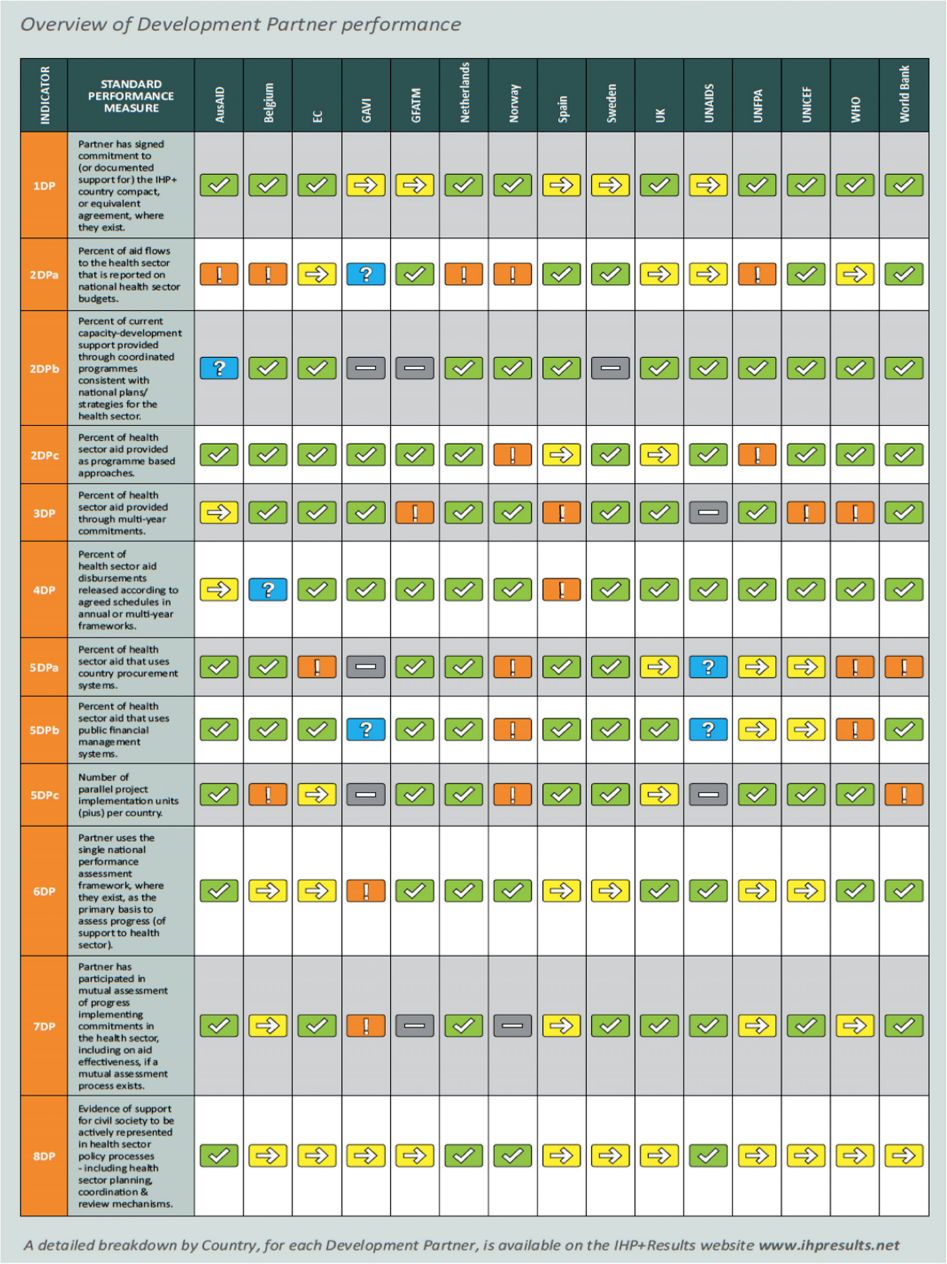
Overview of Development Partner performance.

**Figure 2:**
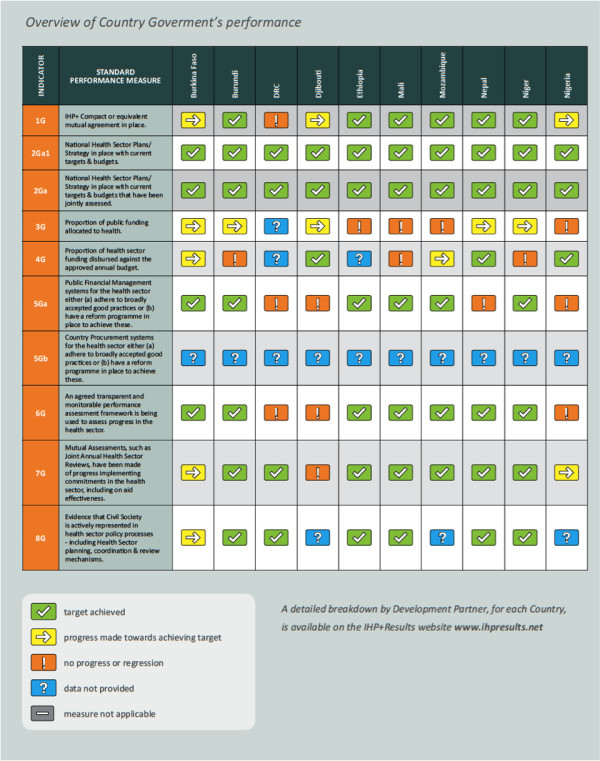
Overview of IHP + Country Government performance.

The following Figures show the Scorecard ratings for the IHP + signatories that participated in *IHP+Results* 2010 monitoring. The following sections draw on and discuss data presented in Figures 1 and 2, and in Table 4 (below). Detailed data are available at http://www.ihpresults.net/data

**Table 4:** Development Partner responses to IHP + country government actions

**Country**	**Compact or Equivalent**		**Performance Framework**		**Mutual Accountability**	
	**Government report**	DP signed*	**Government report**	**DP use**	**Government report**	**DP use**
Burkina Faso		N / A**		8 / 10		N / A
Burundi		8 / 10		6 / 10		5 / 10
DRC		N / A		N / A		3 / 9
Djibouti		N / A		N / A		N / A
Ethiopia		10 / 11		9 / 11		9 / 11
Mali		10 / 10		9 / 10		6 / 10
Mozambique		13 / 14		12 / 14		10 / 14
Nepal		7 / 9		7 / 9		7 / 9
Niger		5 / 9		6 / 9		4 / 9
Nigeria		N / A		N / A		N / A

* Number of Development Partners positively reporting against this indicator over the number of Development Partners that were active in the country and participated in IHP+Results 2010 monitoring.

** N/A: Not Applicable.

The *IHP+Results* monitoring generated data on a range of indicators that we present grouped according to the principles of the Paris Declaration (see above). In our assessment we found that most participating countries and Development Partners had made some progress in improving how they were delivering health aid in line with the Paris principles. Development Partners generally performed well in terms of agreeing to high-level frameworks for delivering aid according to recipient countries’ priorities. However, performance in terms of strengthening and using country systems was below expectations, and performance in providing predictable financing through multi-year commitments was mixed. Countries that experienced the strongest performance by Development Partners (against the agreed reporting framework) were Ethiopia, Mozambique and Mali, while performance was more limited in other countries, some of which were more recent signatories.

#### Country ownership

A key indicator of country ownership is the existence of a ‘country compact’. A country compact is an agreement between government and Development Partners that sets out principles and commitments on how health aid will be managed in support of the national health plan. All IHP + countries reviewed met the target of having a national health plan or strategy in place (2 Ga). By 2009, four countries (Ethiopia, Mali, Mozambique and Nepal) had also agreed a country compact and two (Burundi and Niger) had equivalent agreements in place (1 G). The remaining countries had all expressed an intention to develop country compacts. While a country may have a country compact, not all Development Partners in that country were a signatory. In Ethiopia, Mali, Mozambique and Nepal (1DP) more than 70%^b^ of Development Partners surveyed indicated that they had signed the relevant country compacts. In all countries where the government reports the existence of a compact, the majority of Development Partners reported having signed up to that compact.

Another indicator of country ownership is alignment between Development Partners programs and recipient countries’ national priorities. Findings from the *IHP+Results* monitoring indicate that overall, Development Partners have become more aligned with national priorities: an increasing proportion of health aid is reported on national budgets (2DPa); Development Partners are meeting the agreed target (50%) on providing training and capacity building assistance that is in line with national priorities; and that there is a greater use of a programme-based approach (2DPb) to health aid, rather than the provision of support through stand-alone projects. Across all the countries surveyed, the overall proportion of health aid reported on the national budget increased from 52% at baseline^c^ to 79% in 2009, against a target of 85%. However, only five participating Development Partners actually met this target (the Global Fund; Spain; Sweden; UNICEF and the World Bank), while a further four reported some progress in 2009 compared with their baseline data (EC, UK, UNAIDS, WHO). All Development Partners that provided data acknowledged that they had already met the target of providing 50% capacity building that is aligned with national priorities. Eleven of the 15 Development Partners surveyed reported that they had met the target of 66% of aid delivered through programme based approaches (Australia, Belgium, EC, GAVI, the Global Fund, the Netherlands, Sweden, UNAIDS, UNICEF, WHO and the World Bank).

#### Mutual accountability and managing for results

A single performance assessment framework is deemed central to a government’s efforts to measure health outcomes, monitor progress and identify areas of under-performance. Fragmented performance frameworks and information systems, and high levels of stand-alone project-based monitoring hinder governments’ efforts to have a comprehensive and coherent overview of progress and incurs high transaction costs.

Seven of the ten countries surveyed reported that they had a single national performance framework in place (6 G) while 58% of the Development Partners active in these countries claimed that they were using the national framework as the primary basis to assess the performance of their health aid (6DP) (Table 4). However it was not evident that Development Partners were using the national framework as the sole or primary basis to assess the performance of their own health aid as some reported that they requested additional indicators that went beyond the national framework. *IHP+Results* did not assess the quality of the country performance framework, nor the extent to which the national performance framework covered the private and non-government sectors.

*IHP+Results* also asked countries to report if they had a process of mutual assessment of progress in the health sector (7 G). Seven of ten countries (Burundi, DRC, Ethiopia, Mali, Mozambique, Nepal and Niger) reported having a mutual assessment process in place. The majority of Development Partners participated in these mutual assessment processes in four of the seven countries (7DP), while a minority of Development Partners participated in the process in the other two countries. The Global Fund and Norway consider this not applicable to them while the GAVI Alliance reports not participating.

#### Transparent and responsible financing

There has been mixed progress in securing transparent and responsible health sector financing in the countries monitored. The key indicator for this, for Development Partners, is the percentage of health aid that is committed to countries on a multi-year (three years or more) basis (3DP). Overall the proportion of finance provided by Development Partners through multi-year commitments fell from 75% to 70% between baseline data and 2009, against a target of 90%. However, nine of fifteen Development Partners (Belgium, EC, GAVI, Netherlands, Norway, Sweden, UK, UNFPA and the World Bank) were providing 90% or more of their health aid through multi-year commitments in 2009. When measuring the ability of Development Partners to disburse funds to countries on time (4DP), the survey showed that 95% of Development Partner funding was being disbursed during the year in which this had been committed to the country, compared with 92% at baseline (against a target of 90%).

**Figure 3:**
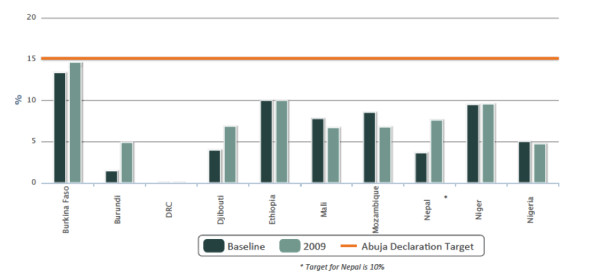
Proportion of national budget allocated to health.

IHP + country government performance on transparent and responsible financing was measured in terms of the proportion of national budgets allocated to health. Five governments (Burkina Faso, Burundi, Djibouti, Nepal and Niger) increased the proportion of their national budgets allocated to health (3G). However these increases were widely variable and none of the African governments monitored had yet met the Abuja target [19] of 15% of national budget allocated to the health sector, although Burkina Faso neared the target with an allocation of 14.6% of the national budget to health in 2009 and Nepal’s domestic spending for health reached 7.6% in 2009 against a national target of 10% (Figure 3).

It is important to note that Figure 3 tracks changes in the allocation of the national budget to health for each surveyed country. However, direct comparisons between countries are difficult because of how external sources of funding have been reported by survey participants. For example, Burkina Faso and Mozambique excluded external assistance in their calculations of the national budget allocated to health, while Djibouti and Niger included external assistance and Burundi, Ethiopia, Mali, Nepal and Nigeria did not provide information on whether they included or excluded external funding in their reported figures.

The performance of governments in disbursing the available health sector budgets also showed a mixed picture (4G). We found that five (Burkina Faso, Djibouti, Mozambique, Nepal and Niger) out of ten countries increased the disbursement in their health budget while three (Burundi, Mali and Nigeria) decreased the proportion of funds disbursed.

#### Using and strengthening country systems

The IHP + encourages Development Partners to use, support and strengthen country systems whilst discouraging the introduction of parallel or stand-alone systems. Two such systems are used as *IHP+Results* indicators: first is the systems used by government to procure goods and services (5DPa), and second is the use of public financial management systems (5DPb) for budgeting, planning and reporting purposes. Both indicators measure the proportion of health aid that is channeled through these government systems. Overall the volume of Development Partner health aid that uses country procurement systems has declined from 60% (multiple agency baselines) to 53% in 2009 (against a target of 80%). Only six Development Partners (Australia, Belgium, the Global Fund, the Netherlands, Spain and Sweden) met the target of a 33% reduction in funds *not* using the national procurement system.^d^ However this result might be underestimated because it does not take into account the fact that a number of Development Partners such as the UK, the EC, the Netherlands, Norway and Sweden provide considerable budget support that would use country procurement systems. The measure is not applicable for the GAVI Alliance whose procurement is conducted through a supra-national pooling mechanism administered by UNICEF. There is also some variation from country to country with, for example, a higher proportion of heath aid was being channeled through country systems in Ethiopia and Mali.

The third indicator of whether or not Development Partners use recipient countries’ national systems is the use of stand-alone project implementation units (PIUs) to manage their health aid (5DPc). The number of such stand-alone PIUs in the ten surveyed countries was reduced by 29% between in 2009, compared to the baseline. This represents significant progress towards achieving the target of a two-thirds reduction. UNFPA reported the largest reduction in the use of PIUs from 21 to six. However, three Development Partners) Belgium, Norway and the World Bank) reported a small increase in the number of PIUs.

IHP + signatory countries have committed to improving both their procurement and public financial management systems. There were insufficient data to determine whether procurement systems were improving within countries so we could not report on this indicator. The World Bank’s rating of overall (non-health-specific) national public financial management systems gives some indication of general trends in aid. The data suggest that three countries (Burkina Faso, Burundi and Mozambique) had improved their public financial management systems while two (Mali and Nepal) had seen a deterioration between 2005 and 2009. Five countries were considered to adhere to ‘good practices’ as defined by the World Bank (Burkina Faso, Ethiopia, Mali, Mozambique and Niger)^d^ . In these five countries 63% of Development Partner funding was reported to have used country financial management systems in 2009, an increase of 18% over the baseline.

#### Development Partners performance

No single Development Partner met all of the selected targets in all of the countries. However, overall, more targets were met than were not met. The Netherlands recorded the highest number of targets met (eleven out of twelve) followed by World Bank (nine) and Sweden (eight). (Australia has also met the targets for eight indicators but this it only delivers health aid to one of the ten countries surveyed). Only two Development Partners reported achieving the target or making progress on all the indicators: Sweden and the UK (although not necessarily in all countries). On the whole, Development Partners reported progress. All Development Partners reported no progress on two or less targets except for WHO (three targets with no progress) and Norway (five targets with no progress). However, further investigation found that Norway’s apparent poor performance was partly related to Norway’s emphasis on channeling aid through multilateral channels rather than bilateral ones, and to its recent shift from health projects and sector budget support to providing more general budget support. Such a change should reflect an improved performance score. But limitations in the evaluation methodology on how to appropriately assess budget support against the full set of *IHP+Results* indicators contributed to Norway scoring poorly. An overview of these results is presented in Figures 1 and 2 (above).

Development Partners have improved the effectiveness of their health aid most of all in Ethiopia, Mali and Mozambique, followed by Nepal and Burkina Faso. There has been less improvement in Burundi, Djibouti, DRC, Niger and Nigeria. Detailed disaggregated ratings for each participating country can be found at http://www.ihpresults.net/results/data

Most Development Partners provided sufficient data to measure progress against all the targets. The two main exceptions were GAVI and UNAIDS. GAVI reported that three targets were not applicable and data were not available for two of the targets. UNAIDS reported that two targets were not applicable and data were not available for an additional two targets. Australia and Belgium each indicated one target for which data were not available, and the Global Fund (two), Netherlands (one), Sweden (one) reported targets that were not applicable to their way of delivering aid.

#### Country Government Performance

Six countries reported having a country compact, a results framework and a mutual accountability process. These included early IHP + signatories, Ethiopia, Nepal, Mali, Mozambique and Burundi, as well as a more recent signatory, Niger.

Five countries reported having achieved seven of the nine targets for which data were available: Burundi, Ethiopia, Mali, Nepal and Niger; and Mozambique reported having achieved six targets. The remaining countries met four (Burkina Faso and DRC) or three (Nigeria and Djibouti) of the targets. Country governments performed best at having a national health plan in place (all ten countries) followed by having an agreed performance framework in place (seven countries). At the other end of the scale, no countries met the target for the proportion of public funding allocated to health, and only three countries (Djibouti, Nepal and Niger) met the target for disbursing their approved annual health budget.

## Discussion

While this paper has been structured around the *IHP+Results* process, it raises a number of broader issues such as the future of the IHP + itself and the role of independent evaluation and performance ratings in promoting positive behaviour change amongst Development Partners and recipient governments.

As far as the findings of *IHP+Results* are concerned, the first question to consider is whether they say anything useful. The answer to this is a qualified ‘yes’. The formal and structured process of monitoring and measuring certain indicators has enabled a number of useful and authoritative conclusions about implementing the Paris agenda within the health sector to be made. For example, we believe that *IHP+Results* has been able to describe and measure some incremental improvements in the strengthening of national planning processes and of principles around mutual accountability (through the global compact and country compacts). It has also shown some measurable progress in Development Partners aligning their support with national budgets and making greater use of programme based approaches.

It has also documented a lack of progress in the use of country-based financial management and procurement systems and in the reduction of duplicative and parallel performance reporting frameworks and information systems. These findings, by virtue of being measured through a systematic and structured process, can be a powerful catalyst for ensuring further improvement in the way that health aid is harnessed to support health improvement and health systems development.

However, there are also several limitations and weaknesses associated with the work of *IHP+Results*. First, the findings only cover those Development Partners and countries who agreed voluntarily to participate in the evaluation (selection bias).

Second, the data used to generate the measurable indicators for each actor were submitted by the actors themselves (information bias) and were not independently verified.

Third, the indicators used by *IHP+Results* were not comprehensive or exhaustive, a weakness that applies to the indicators used to evaluate the Paris Declaration more generally. For example, the evaluation of the Paris Declaration does not include measures of the transaction costs involved in countries applying for funding from different sources or of having to set up multiple country level governance structures. Neither do they cover the important issue of the transparency of funding decisions and disbursements.

Fourth, the methods employed by *IHP+Results* to measure performance are new and require ongoing refinement and improvement. Some of the indicators selected were found to be less useful than anticipated. For example, on the provision of coordinated capacity development/technical assistance (2DPb) and engagement of civil society (8 G/8DP). And as explained previously, the methodology does not fully capture progress when donors shift to general budget support from project-based modalities or sector budget support. Finally, mainly due to resource constraints, the evaluation methodology did not include the qualitative data necessary to interpret and understand the quantitative scores and ratings - for example, where we collected data on the existence of a policy framework, it was not accompanied by any description or measure of the quality of that performance framework. Similarly, concepts like ‘mutual accountability’ are complex and multi-dimensional and cannot be fully understood or measured through a set of quantitative indicators without some contextual and qualitative analysis.

These limitations and weaknesses should not be viewed as a criticism of *IHP+Results*. Rather they reflect the inherent difficulty of rigorously and usefully monitoring the behaviour of multiple institutions operating across multiple countries within a complex system. It also reflects a lack of available and reliable data which is a weakness that needs to be addressed by Development Partners and governments, rather than by an independent evaluator such as *IHP+Results*.

In order to improve the prospects for verifying self-reported data in future, *IHP+Results* will take steps to situate future data collection as an in-country process and also encourage a discussion of draft results by in-country stakeholders. The engagement of local academic organisations or other civil society organisations in verifying data will therefore be critical. A number of additional methodological and process amendments were discussed and agreed with IHP + signatories (through the IHP + working group on mutual accountability) to be implemented in the ongoing 2012 IHP + monitoring exercise (taking place from February to July, and with a report expected in September 2012). These issues include – providing clearer definition on key terminology (including when a Development Partner is ‘active’ in a country and should therefore provide data); and stronger measures of civil society engagement. Participation is also strengthened with 15 Development Partners and 19 countries opting to complete IHP + of monitoring in 2012 (up from 10 in 2010); broadly the twenty-five participants as in 2010 are repeating the exercise.

Notwithstanding these changes and the need for methodological improvements and better data, it is important to note the ground-breaking nature of *IHP+Results*. For the first time, a structured, independent and transparent evaluation of the performance and behaviours of both Development Partners and country governments has been conducted; and in a way that allows for comparisons between actors as well as comparisons over time.

While the validity of the methods and data used to evaluate aid effectiveness and institutional behaviour is important, the *process* of measurement *and* the *publication* of results are equally important and are a vital part of improving mutual accountability. In theory, the adoption of a Scorecard approach will help change behaviour through peer pressure, public scrutiny and increased transparency of any gap between the rhetoric and practice of aid effectiveness. This should also help shift the balance of power between governments, donors and civil society organisations in ways that will improve alignment, harmonisation and health systems strengthening.

However, in moving forward, there is a need to expand the scope and coverage of the monitoring. Not all IHP + signatories opted to participate in the *IHP+Results* process. Notable absentees included the Bill and Melinda Gates Foundation. The biggest bilateral donor, the US government, is not even a signatory of the IHP+. These two absentees, particularly because of their significant influence leave a significant gap in any analysis of the mutual accountability of development partners and recipient countries within the health sector.

The Busan Outcome Document signals a shift from ‘aid effectiveness’ to what has been called ‘development effectiveness’ in which a greater emphasis is placed on results and on the role of non-government actors/donors as well as public-private partnerships. It also signals a greater focus on the outcomes of aid rather than on the process of managing and transferring aid. In many ways, the health sector is ahead of the game because the IHP + global compact already stresses results and has been signed by private actors (Bill and Melinda Gates Foundation) and the major global health partnerships (the GAVI Alliance and Global Fund). The agreement at Busan, which emphasised transparency and accountability, also heightens the relevance of IHP+, not just for the health sector but also for other sectors that could learn from the IHP + experience. However, Busan does make a shift from Paris in diluting commitment and attention to alignment and harmonisation.^d^

The Busan Outcome Document also reflects the continued importance of country ownership and that much work remains to be done in this area. This has been a focus for IHP+. An aim of the IHP + should be to support the development and implementation of tools and instruments which will help foster better quality national plans and strengthen country ownership, and increase Development Partner alignment towards country-owned plans and strategies. Notable amongst these is the Joint Assessment of National Strategies (JANS) approach which seeks to develop a shared method of assessing and strengthening national health strategies, thereby increasing partner confidence in those strategies, securing more predictable and better aligned funding, and reducing transaction costs arising from multiple separate agency assessments.

The value of an independent and external review of progress is clear from the experience of *IHP+Results*. The experience has underlined the importance of on-going monitoring of aid effectiveness, which at the time of writing is unclear as the new set of post-Busan indicators will be agreed later in 2012. Busan signalled increased importance of transparency and accountability in development which requires data in the public domain, and on-going monitoring. This is what *IHP+Results* does. The *IHP+Results* experience can form the basis for on-going improvements in the coordination, implementation and effective use of development assistance for health. It is important that the progress in evaluation reflected in the findings reported here is built upon. Valuable investments have been made in developing a methodology and evaluation instruments that could be built upon.

Any future monitoring of IHP + will need to be affordable and feasible. But it will be important to recognize the trade-off between trying to achieve this and ensuring a comprehensive set of both qualitative and quantitative data. The key factors that seem to enable a more viable long term process include - strong country ownership, agreed reporting frameworks and process, explicit commitments, voluntary reporting, the perception that the principal purpose of the monitoring process is to provide credible data and analysis to feed ongoing dialogue between committed partners.

For a long time the development community has been emphasising the importance of country ownership and leadership. While the Paris Declaration and IHP + have strengthened both country ownership and leadership translating these principles into better systems and better results needs Development Partners to stay the course.

## Conclusion

The global financial crisis is going to reduce aid spending and has led to an increased focus on results and value for money. In light of the sustained (if not growing) need to fund health programs, it seems even more important that aid is used effectively to maximise the achievement of health goals. The findings of this study suggest that the IHP + is making a positive contribution to improving aid effectiveness in the health sector (at least in the ten countries where the study was completed, and for those fifteen Development Partners that participated) in strengthening country ownership, harmonisation and alignment. However progress is varied, both across countries and between and within Development Partner organizations, and it seems clear that there are particular challenges associated with strengthening and using country systems, which suggest that this is a necessary focus for the IHP + going forward.

External, independent monitoring, including *IHP+Results*, has the potential to help strengthening accountability in the health sector, and beyond. But there is a clear need to ensure that civil society organisations have the capacity to engage in a meaningful way. Whilst the investments made in *IHP+Results* methodology and tools have shown that sector level aid effectiveness monitoring can provide credible results, and can generate useful findings, there are also limitations with IHP + monitoring that need to be addressed. This is not surprising given the challenge of rigorously monitoring multiple Development Partners across multiple countries within a complex system. The findings presented here suggest that the health sector is ahead of the game – in terms of having an established mechanism to promote alignment and harmonisation, and having a relatively advanced monitoring framework and methods. But to capitalise on this advantage IHP + signatories should reaffirm commitment to the IHP+, actively participate in monitoring and evaluation processes and strengthen in-country capacity notably amongst civil society organizations.

## Endnote

^a^http://www.aideffectiveness.org/busanhlf4/images/stories/hlf4/OUTCOME_DOCUMENT_-_FINAL_EN.pdf

^b^In a number of instances we refer to a % of Development Partners. This is because the denominator (the number of Development Partners active in each country) varies and the numerator (achievement of a target by Development Partner in a country) is not consistent because some development partners achieve a target in one country and not in another. The % aggregates the findings for all the countries

^c^Baseline data covered a series of years (2005–2007), depending on the data that respondents were able to provide

^d^There are essentially two targets for Development Partner use of both national procurement and public financial management systems: a reduction in funds not using those systems, and an absolute target for the proportion of funds using those systems. For more information on the Paris Declaration targets (which are the basis of *IHP+Results* targets) see http://www.oecd.org/dataoecd/57/60/36080258.pdf

^e^Data (and therefore ratings) on the strength of country procurement and public financial management systems were taken from existing sources: for procurement Paris Declaration monitoring data was used where available; for public financial management systems, World Bank data Country Policy and Institutional Assessment (CPIA)/PFM data were used. Whether a country ‘adhered to accepted good practices’ was determined by the OECD and World Bank scores shown in these data, and the Paris Declaration targets. For example, if a country’s PFM system scored less than 3.5 the Paris Declaration target includes no explicit expectation that Development Partners will channel their funding through that system; the comparable score for country procurement systems was B (on a four-point scale A-D).

^f^See Busan Outcome Document for more details: http://www.aideffectiveness.org/busanhlf4/images/stories/hlf4/OUTCOME_DOCUMENT_-_FINAL_EN.pdf

## Competing interests

*IHP+Results* annual, independent monitoring is funded through a contract with the World Health Organization, on behalf of IHP + signatories. TS (as Project Manager) and MT were contracted to implement *IHP+Results* methodology and subsequent analysis under the WHO contract (described above). NS and SMJ were also contracted, albeit to a lesser extent, under the *IHPResults* contract with WHO. DM is a member of *IHP+Results* Independent Advisory Group (IAG), and received an honorarium funded by the *IHP+Results* WHO contract for his role in the IAG.

## Authors’ contributions

NS led on drafting the Background section, TS led on drafting the Methods section, MT and SMJ led on drafting the Results section, and DM led on drafting the discussion section. All authors were involved in commenting on and finalising the final draft. The study conception, design and execution – including analysis and interpretation of data – was undertaken by the *IHP+Results* consortium, to which TS, NS, SMJ formally belong, and MT provided substantial (lead author) contributions. All authors read and approved the final manuscript
